# Donor insulin therapy in intensive care predicts early outcomes after pancreas transplantation

**DOI:** 10.1007/s00125-021-05411-9

**Published:** 2021-03-04

**Authors:** Iestyn M. Shapey, Angela Summers, Hussein Khambalia, Petros Yiannoullou, Catherine Fullwood, Neil A. Hanley, Titus Augustine, Martin K. Rutter, David van Dellen

**Affiliations:** 1 Faculty of Medicine, Biology and Health, University of Manchester, Manchester, UK; 2 Department of Renal and Pancreatic Transplantation, Manchester University NHS Foundation Trust, Manchester Academic Health Science Centre, Manchester, UK; 3 Department of Research and Innovation (Medical Statistics), Manchester University NHS Foundation Trust, Manchester Academic Health Science Centre, Manchester, UK; 4 Manchester Diabetes Centre, Manchester University NHS Foundation Trust, Manchester Academic Health Science Centre, Manchester, UK

**Keywords:** Insulin, Islet, Organ donor, Pancreas, Transplant

## Abstract

**Aims/hypothesis:**

Approximately 50% of organ donors develop hyperglycaemia in intensive care, which is managed with insulin therapy. We aimed to determine the relationships between donor insulin use (DIU) and graft failure in pancreas transplantation.

**Methods:**

UK Transplant Registry organ donor data were linked with national data from the UK solid pancreas transplant programme. All pancreas transplants performed between 2004 and 2016 with complete follow-up data were included. Logistic regression models determined associations between DIU and causes of graft failure within 3 months. Area under the receiver operating characteristic curve (aROC) and net reclassification improvement (NRI) assessed the added value of DIU as a predictor of graft failure.

**Results:**

In 2168 pancreas transplant recipients, 1112 (51%) donors were insulin-treated. DIU was associated with a higher risk of graft loss from isolated islet failure: OR (95% CI), 1.79 (1.05, 3.07), *p* = 0.03, and this relationship was duration/dose dependent. DIU was also associated with a higher risk of graft loss from anastomotic leak (2.72 [1.07, 6.92], *p* = 0.04) and a lower risk of graft loss from thrombosis (0.62 [0.39, 0.96], *p* = 0.03), although duration/dose-dependent relationships were only identified in pancreas transplant alone/pancreas after kidney transplant recipients with grafts failing due to thrombosis (0.86 [0.74, 0.99], *p* = 0.03). The relationships between donor insulin characteristics and isolated islet failure remained significant after adjusting for potential confounders: DIU 1.75 (1.02, 2.99), *p* = 0.04; duration 1.08 (1.01, 1.16), *p* = 0.03. In multivariable analyses, donor insulin characteristics remained significant predictors of lower risk of graft thrombosis in pancreas transplant alone/pancreas after kidney transplant recipients: DIU, 0.34 (0.13, 0.90), *p* = 0.03; insulin duration/dose, 0.02 (0.001, 0.85), *p* = 0.04. When data on insulin were added to models predicting isolated islet failure, a significant improvement in discrimination and risk reclassification was observed in all models: no DIU aROC 0.56; DIU aROC 0.57, *p* = 0.86; NRI 0.28, *p* < 0.00001; insulin duration aROC 0.60, *p* = 0.47; NRI 0.35, *p* < 0.00001.

**Conclusions/interpretation:**

DIU predicts graft survival in pancreas transplant recipients. This assessment could help improve donor selection and thereby improve patient and graft outcomes.

**Graphical abstract:**

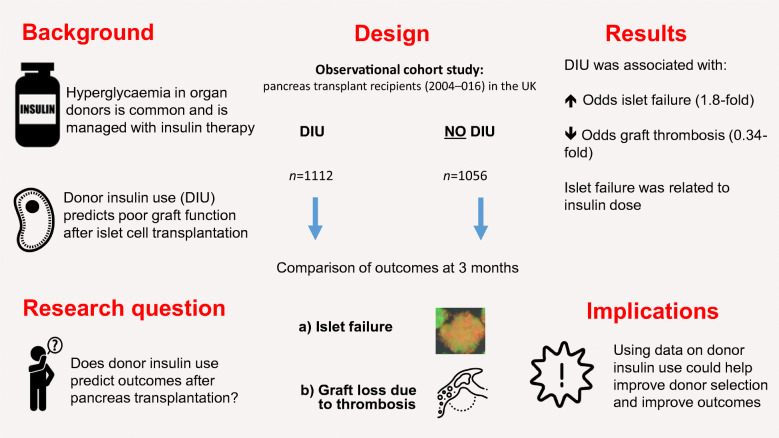

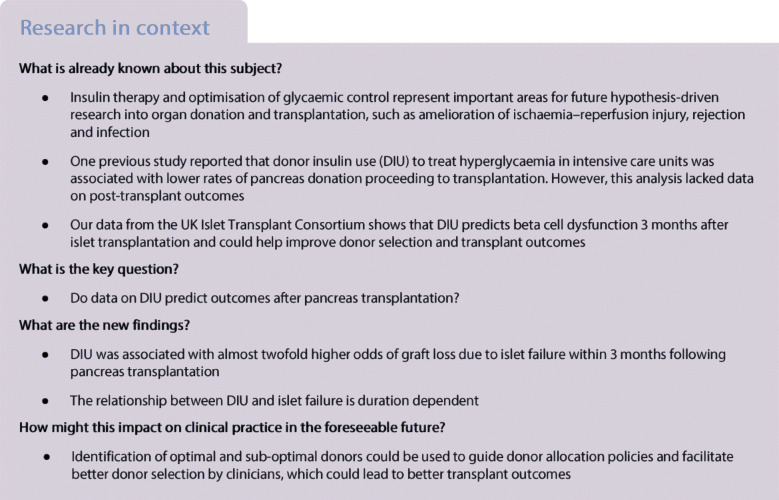



## Introduction

Diabetes mellitus is a disease associated with high risks for morbidity and mortality due to multi-system micro- and macro-vascular complications. Pancreas transplantation (simultaneous pancreas and kidney, pancreas after kidney or pancreas transplant alone) offers a highly effective and life-saving therapy for patients with severe hypoglycaemia or renal failure due to diabetes mellitus [1].

Inappropriate donor organ selection impacts adversely on patient outcomes in pancreas transplantation [2–4]. There is an urgent need to identify robust objective methods for the assessment and selection of high-quality pancreases [5]. However, objective measurement of organ quality remains a significant challenge. The pancreas donor risk index (PDRI) [4], a composite score calculated from donor factors, is the best available assessment tool, but can provide unreliable results [6, 7].

The pancreas is extremely sensitive to ischaemia and injury as a consequence of brain death or organ procurement [8, 9]. In ~50% of donors, corticosteroids are given to counter the inflammatory response to brain death. Steroid administration is associated with donor hyperglycaemia [10]. However, the combined insults of systemic inflammation, catecholamine surge and metabolic stress could also contribute to donor hyperglycaemia. Insulin, given in intensive care units (ICUs) in response to donor hyperglycaemia, may modulate the inflammatory, metabolic and thrombotic responses to brain death as well as controlling glucose levels [11–13].

In an analysis of registry data, Novitzky et al reported that donor insulin use (DIU) to treat hyperglycaemia in ICUs was associated with lower rates of pancreas donation proceeding to transplantation. The authors speculated that DIU could be a marker of donor pancreatic failure [14]. However, this analysis lacked data on post-transplant outcomes [14]. We have previously demonstrated that DIU predicts poorer islet function following islet transplantation [15]. Therefore, we hypothesised that DIU is a predictor of adverse outcomes in pancreas transplantation and aimed to assess the relationships of DIU with graft failure.

## Methods

### Patient cohorts

Data on all organ donors and recipients from the UK solid organ pancreas transplant programme (2004–2016) were accessed from the UK Transplant Registry, held by NHS Blood and Transplant (NHSBT). We included all cases of pancreas transplantation from the registry which commenced in 2004. Data were collected prospectively with written informed consent from all transplant recipients with type 1 diabetes mellitus.

### Standardising DIU

The UK NHSBT donor care bundle provides standardised guidance to maintain donor glucose levels between 4 and 10 mmol/l and to start a variable rate insulin infusion (VRII) at a minimum rate of 1 U/h when appropriate [16]. DIU was defined as any requirement for exogenous insulin during the peri-donation period. The duration of insulin use was also considered, but data on total insulin dosage were not available.

### Defining graft failure

Early pancreas graft failure was defined as a return to exogenous insulin therapy within 3 months post transplantation. Pancreas graft failures from all causes were considered in the analysis and included thrombosis, pancreatitis, anastomotic leak, infection/sepsis, rejection, bleeding, islet failure with no attributable cause and death with functioning graft. Kidney graft failure was defined as a return to dialysis dependence.

### Statistical methods

We assessed the distribution of donor and recipient variables for exposures, covariates and outcomes. Cases with missing outcome data (*n* = 103) were immediately excluded. Data on cold ischaemic time were missing in 9% of cases, for donor creatinine in 12% of cases and for recipient BMI in 27% of cases; this was addressed by using pooled results from multiple imputation. Missing data for all other variables were <1%. Covariates were compared by DIU status using Fisher’s exact test, Student’s *t* test or the Mann–Whitney *U* test, as appropriate. Univariable and multivariable logistic regression models determined relationships between DIU and graft failure within 3 months of transplantation. Follow-up began at transplantation and ended 3 months post transplantation. Multivariable models included potential confounders that were identified at univariable analysis (*p* < 0.1). Receiver operating characteristic curves (ROCs) were calculated for all predictive models. Net reclassification improvement (NRI) was used to determine the added value of insulin use as a predictor of early graft failure [17].

Statistical analysis was performed using SPSS (IBM SPSS Statistics for Windows, version 22.0. Armonk, NY, USA: IBM Corp, 2013 release). Statistical significance was assumed with *p* < 0.05 unless otherwise stated.

## Results

Of the 2168 pancreas transplant recipients with complete follow-up data (1866 simultaneous pancreas and kidney; 151 pancreas after kidney; 151 pancreas transplant alone; female: *n* = 925 [42.7%]; mean [SD] age: 42 [8] years, BMI 23.5 [3.4] kg/m^2^), 1112 (51%) had a donor treated with insulin; the median (IQR) duration of insulin use was 35 (20–58) h. Table 1 shows that donor characteristics associated with DIU included: donor type (proportion with donor after brain death: DIU vs no DIU, 90% vs 75%; *p* < 0.001); sex (female 54% vs 45%; *p* < 0.001); use of corticosteroids (43% vs 29%; *p* < 0.001); age (35 [13.2] vs 34 [13.8]; *p* = 0.013); cardiac arrest (27% vs 31%; *p* = 0.03); and trauma as cause of death (14% vs 20%, *p* = 0.001).

**Table 1:** Characteristics associated with DIU in intensive care in pancreas donors

Donor variable	DIU in intensive care		*p* value
	Yes (*n* = 1112)	No (*n* = 1052)	
Age, years	35 ± 13	34 ± 14	0.01
BMI, kg/m^2^	24 ± 3.6	24 ± 3.5	0.21
Sex (female)	600 (54)	477 (45)	<0.0001
Ethnicity			
White	1019 (92)	980 (93)	0.27^a^
Asian	25 (2.2)	15 (1.4)	NA
Black	16 (1.4)	14 (1.3)	NA
Other	52 (4.7)	43 (4.1)	NA
Smoking	543 (49)	530 (50)	0.52
Alcohol excess	75 (6.7)	89 (8.5)	0.12
Hypertension	86 (7.7)	99 (9.4)	0.19
Cardiac disease	37 (3.3)	28 (2.7)	0.45
Cardiac arrest	299 (27)	329 (31)	0.03
Donor type (DBD)	1005 (90)	788 (75)	<0.0001
Cause of death			
Trauma	157 (14)	206 (20)	<0.01
Meningitis	39 (3.5)	12 (1.1)	<0.01
Stroke (thrombo-embolic)	64 (5.8)	56 (5.3)	0.71
Intracranial haemorrhage	578 (52.0)	510 (49)	0.11
Hypoxic brain damage	186 (17)	207 (20)	0.08
Brain tumour	16 (1.4)	18 (1.7)	0.73
Other	72 (6.5)	43 (4.1)	NA
Creatinine >221 μmol/l^b^	26 (2.3)	19 (1.8)	0.55
Methylprednisolone use	483 (43)	300 (29)	<0.0001

Age and BMI are continuous data presented as mean±SD. All other data are binary and presented as *n* (%)

All available variables were included in the analysis

Smoking: either past or present

Alcohol excess: ≥7 units/day

Cardiac disease: either ischaemic heart disease or valvular disease

Cardiac arrest: cessation of circulation during the acute event that led to organ donation

Methylprednisolone use: 15 mg/kg to a maximum of 1 g as outlined in the donor care bundle

^a^Ethnicity: white vs non-white

^b^Missing data handled by multiple imputation (12% of cases)

NA, Not applicable; DBD, donation after brain death

There were 261 graft failures within 3 months post transplantation: thrombosis, 83 (32%); pancreatitis, 21 (8%); anastomotic leak, 23 (9%); infection, 21 (8%); bleeding, 23 (9%); rejection, 8 (3%); isolated islet failure with no attributable cause, 60 (23%); and death with functioning graft, 22 (8%). Univariable logistic regression demonstrated that DIU was associated with a higher risk of graft loss from islet failure (OR [95% CI], 1.79 [1.05, 3.07], *p* = 0.03), and that a duration-dependent relationship was seen (1.00 [1.00, 1.01], *p* = 0.02). DIU was also associated with a higher risk of graft loss from anastomotic leak (2.72 [1.07, 6.92], *p* = 0.04) and a lower risk of graft loss from thrombosis (0.62 [0.39, 0.96], *p* = 0.03), although duration/dose-dependent relationships were only identified in pancreas transplant alone/pancreas after kidney transplant (PTA/PAK) recipients with grafts failing due to thrombosis (0.86 [0.74, 0.99], *p* = 0.03). DIU was not a significant predictor of other causes of graft loss within 3 months (Table 2). Neither DIU nor insulin duration/dose was a predictor of kidney graft failure.

**Table 2:** Donor insulin-related graft failure within 3 months after pancreas transplantation in univariable analysis

Cause of failure	*n*	DIU		Donor insulin duration	
		OR (95% CI)	*p* value	OR (95% CI)	*p* value
Thrombosis	83	0.62 (0.39, 0.96)	0.03	0.99 (0.98, 1.01)	0.34
Pancreatitis	21	0.71 (0.30, 1.69)	0.44	1.00 (0.99, 1.01)	0.89
Anastomotic leak	23	2.72 (1.07, 6.92)	0.04	1.00 (0.99, 1.01)	0.45
Infection	21	0.57 (0.14, 2.39)	0.44	0.90 (0.72, 1.12)	0.33
Bleeding	23	1.24 (0.54, 2.83)	0.62	1.00 (0.99, 1.01)	0.45
Rejection	8	2.86 (0.58, 14.20)	0.20	0.89 (0.78, 1.02)	0.09
Isolated islet failure	60	1.79 (1.05, 3.07)	0.03	1.00 (1.00, 1.01)	0.02
Death with functioning graft	22	0.79 (0.34, 1.84)	0.58	1.00 (0.99, 1.01)	0.76

Other nominal predictors of graft loss from islet failure included donor BMI (OR [95% CI], 1.09 [1.01, 1.17], *p* < 0.01) and cold ischaemic time (1.10 [1.02, 1.19], *p* = 0.01), which were included as covariates in appropriate regression models (*p* < 0.1 inclusion threshold) (Table 3). Donor age, donor BMI, donor type (comparing donors after circulatory death with donors after brain death), thrombo-embolic stroke as a cause of donor death, cold ischaemic time and transplant type (simultaneous pancreas and kidney transplantation vs pancreas transplant alone or pancreas after kidney transplant) were predictors of thrombosis as the cause of graft failure (Table 3). Donor age and donor BMI were predictors of failure owing to anastomotic leak. Cold ischaemic time and transplant type were predictors of failure owing to graft rejection (Table 3). Recipient BMI was a weak (but non-significant) predictor of failure due to anastomotic leak (1.10 [0.98, 1.25], *p* = 0.09) and was included as a covariate in the appropriate multivariable regression analysis. Otherwise, recipient age, BMI and sex were not significant predictors of outcomes (Table 3).

**Table 3:** Donor and recipient factor-related graft failure 3 months after pancreas transplantation in univariable analysis

Variables	n/N (%)	Thrombosis		Anastomotic leak		Islet failure (no attributable cause)		Rejection	
		OR (95% CI)	*p* value	OR (95% CI)	*p* value	OR (95% CI)	*p* value	OR (95% CI)	*p* value
Donor									
Age, years		1.02 (1.01, 1.04)	<0.01	1.04 (1.01, 1.07)	0.023	1.02 (0.99, 1.04)	0.13	0.99 (0.94, 1.04)	0.57
BMI, kg/m^2^		1.09 (1.03, 1.16)	<0.01	1.12 (1.00, 1.26)	0.056	1.09 (1.01, 1.17)	0.02	1.00 (0.814, 1.22)	0.97
Sex (female)	1132/2168 (52)	0.79 (0.51, 1.23)	0.29	0.52 (0.22, 1.24)	0.14	0.99 (0.59, 1.65)	0.97	1.65 (0.39, 6.93)	0.49
Hypertension	228/2168 (11)	1.66 (0.86, 3.18)	0.13	0.48 (0.06, 3.57)	0.47	1.66 (0.78, 3.54)	0.19	0	1.00
Donor type (DBD vs DCD)	393/2168 (18)	1.91 (1.16, 3.13)	0.01	1.72 (0.67, 4.38)	0.26	0.53 (0.23, 1.24)	0.14	0.69 (0.09, 5.62)	0.73
Cause of death									
Trauma	374/2168 (17)	0.75 (0.39, 1.43)	0.38	0.47 (0.11, 2.01)	0.31	0.76 (0.36, 1.61)	0.47	1.66 (0.33, 8.24)	0.54
Meningitis	54/2168 (2)	1.03 (0.25, 4.29)	0.97	0	1.00	1.45 (0.34, 6.10)	0.61	6.03 (0.73, 49.9)	0.10
Stroke (thrombo-embolic)	124/2168 (6)	2.74 (1.41, 5.32)	0.01	0	1.00	0.89 (0.27, 2.88)	0.84	2.43 (0.30, 19.90)	0.41
Intracranial haemorrhage	1139/2168 (53)	1.30 (0.84, 2.03)	0.24	1.87 (0.78, 4.42)	0.16	1.40 (0.83, 2.35)	0.21	0.59 (0.14, 2.48)	0.47
Hypoxic brain damage	421/2168 (19)	0.68 (0.36, 1.30)	0.24	1.60 (0.63, 4.09)	0.32	0.69 (0.32, 1.46)	0.33	0	0.99
Brain tumour	35/2168 (2)	0	1.00	0	1.00	0	1.00	0	1.00
Methylprednisolone use	834/2168 (38)	0.71 (0.44, 1.14)	0.16	2.31 (1.01, 5.28)	0.05	1.26 (0.75, 2.13)	0.38	0.59 (0.12, 2.90)	0.51
Recipient									
Age, years		0.98 (0.96, 1.01)	0.12	1.01 (0.96, 1.06)	0.66	1.00 (0.97, 1.03)	0.92	1.07 (0.98, 1.16)	0.12
BMI, kg/m^2a^		0.99 (0.94, 1.04)	0.66	1.10 (0.98, 1.25)	0.09	1.07 (0.99, 1.16)	0.10	0.92 (0.71, 1.19)	0.52
Sex (female)	925/2168 (43)	1.08 (0.74, 1.58)	0.69	0.68 (0.30, 1.55)	0.36	0.97 (0.58, 1.63)	0.92	0.45 (0.09, 2.22)	0.32
CIT, h^a^		1.06 (0.99, 1.13)	0.08	1.07 (0.95, 1.21)	0.28	1.10 (1.02, 1.19)	0.01	1.32 (1.11, 1.57)	<0.01
Transplant type (SPK vs PTA/PAK)	1866/2168 (86)	0.40 (0.25, 0.66)	<0.0001	1.08 (0.32, 3.67)	0.90	0.71 (0.37, 1.39)	0.32	0.10 (0.02, 0.40)	<0.001

Age, BMI and CIT are continuous data; all other variables are binary data. All available variables were included in the analysis

^a^Missing data handled by multiple imputation (CIT 9%, recipient BMI 27% of cases)

Methylprednisolone use: 15 mg/kg to a maximum of 1 g as outlined in the donor care bundle

DBD, donation after brain death; DCD, donor after circulatory death; CIT, cold ischaemic time; SPK, simultaneous pancreas kidney

The relationship between donor insulin and higher likelihood of islet failure remained significant after adjusting for potential confounders (BMI and cold ischaemic time): DIU, 1.75 (1.02, 2.99), *p* = 0.04; duration/dose, 1.08 (1.01, 1.16), *p* = 0.03 (Table 4). Relationships between graft failures owing to anastomotic leak, rejection and thrombosis did not remain significant after adjusting for potential confounders (Table 4). However, when analysed according to sub-groups of transplant type (simultaneous pancreas kidney or PTA/PAK), data on insulin remained as significant predictors of lower risk of graft thrombosis in multivariable analyses: DIU, 0.34 (0.13, 0.90), *p* = 0.029; insulin duration/dose, 0.02 (0.001, 0.85), *p* = 0.040.

**Table 4:** Multivariable analysis for graft failure within 3 months after pancreas transplantation

Cause of failure	Predictor	Insulin use		Predictor	Insulin duration	
		OR (95% CI)	*p* value		OR (95% CI)	*p* value
Islet failure						
	DIU	1.75 (1.02, 2.99)	0.04	Donor insulin duration	1.08 (1.01, 1.16)	0.03
	Donor BMI	1.09 (1.01, 1.17)	0.03	Donor BMI	1.05 (0.95, 1.17)	0.34
	CIT	1.10 (1.02, 1.19)	0.02	CIT	1.13 (1.02, 1.25)	0.02
Thrombosis						
	DIU	0.72 (0.49, 1.07)	0.11	Donor insulin duration	0.87 (0.69, 1.09)	0.22
	Donor age	1.02 (1.01, 1.04)	<0.01	Donor age	1.01 (0.99, 1.04)	0.32
	Donor BMI	1.09 (1.02, 1.15)	<0.01	Donor BMI	1.09 (0.99, 1.21)	0.09
	Donor type (DCD)	1.50 (0.94, 2.41)	0.09	Donor type (DCD)	1.60 (0.63, 4.06)	0.32
	Cause of death (stroke)	2.03 (1.08, 3.81)	0.03	Cause of death (stroke)	3.29 (1.28, 8.45)	0.01
	Transplant type (SPK)	0.35 (0.23, 0.55)	<0.00001	Transplant type (SPK)	0.64 (0.27, 1.53)	0.32
	CIT	1.05 (0.99, 1.12)	0.09	CIT	1.08 (0.97, 1.19)	0.17
Anastomotic leak						
	DIU	2.91 (0.93, 9.10)	0.07	Donor insulin duration	1.00 (1.00, 1.01)	0.36
	Donor age	1.01 (0.97, 1.05)	0.61	Donor age	1.02 (0.97, 1.08)	0.36
	Donor BMI	1.13 (0.97, 1.30)	0.11	Donor BMI	1.14 (0.94, 1.37)	0.19
	Recipient BMI	1.11 (0.98, 1.26)	0.10	Recipient BMI	1.16 (0.98, 1.37)	0.08
Rejection						
	DIU	3.10 (0.61, 15.69)	0.17	Donor insulin duration	0.04 (0.00, 1.49)	0.08
	Transplant type	0.11 (0.03, 0.045)	<0.01	Transplant type	0.08 (0.01, 0.95)	0.05
	CIT	1.29 (1.08, 1.54)	<0.01	CIT	1.34 (0.96, 1.85)	0.08

Transplant type is defined as simultaneous pancreas kidney vs pancreas alone and pancreas after kidney

Age, BMI and CIT are continuous data; all other variables are binary data

CIT, cold ischaemic time; DCD, donor after circulatory death; SPK, simultaneous pancreas kidney

When data on insulin were added to models predicting isolated islet failure, a significant improvement in risk reclassification (NRI) was observed in all models: without insulin, area under the receiver operating characteristic curve (aROC) 0.56; DIU, aROC 0.57, *p* = 0.86; NRI 0.28, *p* < 0.00001; insulin duration, aROC 0.60, *p* = 0.47; NRI 0.35, *p* < 0.00001 (Table 5).

**Table 5:** Improvements in outcome discrimination and risk reclassification for isolated islet failure associated with adding donor insulin to base prediction models

Variables in the predictive models	aROC	aROC (SEM) improvement	*p* value	NRI	*p* value
BMI + CIT	0.56	Reference		Reference	
BMI + CIT + insulin use	0.57	0.01 (0.06)	0.856	0.28	<0.00001
BMI + CIT + insulin duration	0.60	0.04 (0.05)	0.468	0.35	<0.00001

*p* values refer to the assessment of aROC improvement or NRI associated with adding DIU to models

BMI and CIT are continuous data; all other variables are binary data

All stated variables are included in the models

CIT, cold ischaemic time

## Discussion

### Main findings

We have shown that: (1) DIU was associated with 1.79-fold higher odds of graft loss due to islet failure within 3 months following pancreas transplantation; (2) the relationship between DIU and islet failure is duration/dose dependent; and (3) data on DIU and insulin duration/dose improve the performance of models to predict post-transplant islet failure. We have also shown that risk of graft thrombosis in recipients of PTA/PAK was threefold lower when pancreases were transplanted from donors that received insulin compared with from donors not receiving insulin, and that this relationship was dependent on the duration/dose of insulin.

### Prior studies

In 63,593 brain-dead donors in the United Network for Organ Sharing registry, Novitzky et al assessed the relationships between donor use of several hormones in intensive care (thyroid hormone, antidiuretic hormone, corticosteroids and insulin, and their combinations) and the procurement of multiple organs (heart, lungs, kidneys, liver, pancreas, intestine) for transplantation [14]. The authors reported that across all hormone treatment combinations, DIU, compared with no DIU, was associated with a 16% lower (15% vs 18%) use of organs for pancreas transplantation, although no reason for non-transplantation was provided. In four out of eight hormone treatment combinations involving insulin in intensive care, the lower rates of use of organs for pancreas transplantation were statistically significant, although only two of these comparisons would have remained significant after adjusting for multiple comparisons. Whilst their analysis lacked data on post-transplantation function or outcomes, the authors speculated that DIU could either be directly harmful to pancreatic beta cells or could simply be a marker of donor pancreatic failure [14]. However, since pancreatic endocrine function is not formally monitored in ICUs, it is difficult to understand how DIU could lead to lower rates of organ use, unless surgeons were rejecting organs simply based on an assumption that DIU was a marker of beta cell dysfunction. Another important limitation of the study is that the authors were unable to exclude pre-existing diabetes in ‘many’ of the donors. Therefore, it is possible that surgeons were appropriately rejecting pancreases from donors treated with insulin in ICUs because they had diabetes.

We have previously reported data from the entire UK experience of islet transplantation, where we showed that DIU was a significant predictor of islet function 3 months post transplantation [15]. Specifically, we demonstrated the relationships of DIU with higher HbA_1c_ and fasting and 90 min stimulated glucose, lower fasting C-peptide and a lower BETA-2 score (a validated composite measure of graft function) [15, 18]. Higher rates of graft failure were also seen in transplants with DIU, although this relationship was not statistically significant, in part because of the impact of relatively small numbers on the power of the study. In pancreas transplantation, islet failure that is not associated with any discernible underlying cause is the category of graft failure that is most closely aligned with graft failure and impaired function in islet transplantation. It is reasonable to consider, therefore, that the data relating to islet failure following pancreas and islet transplantation are concordant.

### Mechanistic insights

We hypothesised that DIU would be a predictor of adverse outcomes in solid pancreas transplantation. However, we showed that DIU had contrasting relationships with graft failure according to the underlying cause. This poses numerous challenges in interpreting the data, both in the context of existing literature and known bio-molecular pathways as well as in identifying new and potentially plausible mechanistic explanations.

Beta cell stress and insulin resistance is a clearly documented phenomenon that occurs after brain death and which may be accountable for the need for insulin to manage hyperglycaemia following brain death [19]. It is reasonable to speculate that isolated islet failure, as a cause of graft loss, may be due to beta cell death, either as undiagnosed pre-existing diabetes mellitus or as a consequence of the high levels of inflammation and inotrope and corticosteroid administration during organ donation. Yet, in our data on islet transplantation, we demonstrated that DIU was associated with similar HbA_1c_ values to donors not treated with insulin, thereby excluding the pre-existing diabetes hypothesis [15]. The hypothesis that beta cell death occurring secondarily to brain death may be accountable for a sizeable proportion of early graft losses following pancreas and islet transplantation is worthy of due consideration and further research.

In our data, there was no relationship between DIU and graft loss from pancreatitis. One novel explanation is that insulin, once given (because of beta cell failure and/or stress), could exert a protective effect upon pancreatic acinar cells (the predominant cell type in pancreas transplantation). In animal models of acute pancreatitis, insulin-treated pancreatic acinar cells are protected against cytosolic calcium overload and cellular death [20]. This cellular protection could be mediated by insulin promoting glycolytic metabolism, preventing ATP depletion and maintaining the plasma membrane calcium pump [21]. Interestingly, we showed no relationship between the duration of insulin use and graft failure.

The relationship between DIU and lower risk of graft thrombosis is interesting, not least because insulin therapy is known to have potent anti-inflammatory and anti-thrombotic properties [11, 22]. Peri-transplant insulin–heparin infusion has been reported to be associated with enhanced islet survival, although this has not been demonstrated in pancreas transplantation [23]. We speculate that insulin therapy in the donor leads to a reduction in ischaemia–reperfusion injury and therefore better survival outcomes. Reperfusion pancreatitis represents the predominant manifestation of ischaemia–reperfusion injury, and is the precipitating event associated with early graft loss from thrombosis, rejection and sepsis [24–30]. Inter-cellular adhesion molecule-1 (ICAM-1)-mediated micro-circulatory failure is the underlying pathophysiological process resulting in reperfusion pancreatitis [26, 31–33]. Intensive insulin therapy in critically ill patients has been demonstrated to reduce morbidity and mortality [34], and clinical benefits have been attributed to the anti-inflammatory and metabolic properties of insulin and not merely to improvements in glycaemic control per se [13]. Intensive insulin therapy has been shown to lower levels of circulating ICAM1 and nitric oxide, both of which act on the endothelium [35]; this mechanism could also explain the lower rates of graft failure from thrombosis in donors receiving insulin.

### Clinical implications

Identification of the optimal and sub-optimal donor has implications for both the selection and the allocation of donor pancreases. Low utilisation of existing predictive models by clinicians to guide donor selection [4, 36] can be attributed, in part, to the fact that validation studies have not uniformly confirmed the predictive value of risk scores [6, 7, 37–39]. We have shown the value of including data on DIU in predicting outcomes following both modalities of beta cell replacement therapy. DIU predicts a higher risk of isolated islet failure and a lower risk of thrombosis and this ought to be considered in the context of other predictors of adverse outcomes when selecting donors. For example, transplant type was a significant predictor of graft thrombosis but not of isolated islet failure. Therefore, selection of donors using insulin for PTA/PAK transplants, where a significant reduction in thrombosis risk was seen with DIU, may offset the higher risk of thrombosis normally seen in PTA/PAK recipients. Likewise, selecting older donors using insulin might be appropriate when the increased risk of isolated islet failure may be considered an acceptable trade-off in exchange for a lower risk of graft thrombosis.

Routine measurement of HbA_1c_ in potential donors would help to identify those with pre-existing but unidentified diabetes mellitus and may be a useful adjunct to prevent islet failure in recipients post transplantation.

### Research implications

Further research is called for to determine whether there is a causal relationship between DIU and outcomes in pancreas transplantation. This might be best achieved within a randomised controlled trial of insulin therapy in organ donors in intensive care. However, performing such research in deceased people, or patients with no capacity and in whom on-going clinical care has been deemed futile, within legal restrictions poses several practical, clinical and ethical challenges. One such challenge is the standardisation of the management of potential organ donors in intensive care, in particular the indications for and duration of insulin use. In the meantime, animal models provide an opportunity to explore the mechanistic processes underlying the improved outcomes in pancreas transplantation associated with DIU.

In donors who received both corticosteroids and insulin therapy, corticosteroids were administered before the initiation of insulin in only 78 (3.6%) donors. Interestingly, this confirms that administration of high-dose corticosteroids is an unlikely cause of donor hyperglycaemia necessitating insulin therapy. Further research is required to investigate the mechanistic relationships among donor hyperglycaemia, insulin therapy and beta cell function in both donors and transplant recipients.

The modest aROC (0.60) achieved by our modelling using data on donor insulin demonstrates that the potential for robust donor selection based on donor characteristics alone is limited. Our research highlights the potential value in improving objective assessment of donor pancreas quality, including providing better measures of pancreatic cellular physiology prior to organ retrieval. Exclusion of donors exhibiting high levels of cellular abnormality, such as apoptosis or inflammation, could appropriately remove those organs from the donor pool because they could be predicted to fail early. This could spare recipients unnecessary surgical risk, antibody sensitisation and anxiety, and provide recourse and cost benefits to healthcare systems. Such assessment could also help to expand the potential donor pool by using organs with low levels of cell death from donors currently deemed unsuitable based on donor characteristics.

### Strengths and limitations

Using data from the entire UK cohort of pancreas transplantation, we provide the first analysis of the relationship between DIU and clinical outcomes. The demonstration of a significant duration/dose-dependent relationship between DIU and graft loss from islet failure validates the findings of our previous data in islet transplantation [15].

We acknowledge some limitations of this study: First, our study is subject to the limitations associated with the observational retrospective design of registry studies, although this can be offset by robust data capture. This is highlighted in that data on total insulin dosage are not routinely collected, and we had only categorical data on insulin use and continuous data on insulin duration. Further retrospective studies to validate our findings would likewise be subject to the same issues, not least because different institutions in different countries may have different indications for insulin use in ICUs. Second, there is the potential for unmeasured confounders that could explain the association between DIU and outcomes, although all conventional covariates were included and accounted for. Finally, we are unable to determine causality, but the findings are highly valuable in generating hypotheses to test in future work.

### Conclusions

We provide data showing that DIU is associated with a higher risk of graft loss due to islet failure and a lower risk of graft loss due to thrombosis in pancreas transplant recipients. Our research highlights the potential value of developing an objective assessment of donor pancreas quality including a reliable measure of pancreatic physiology prior to organ retrieval. This assessment could help to improve donor selection and thereby improve patient and graft outcomes.

## Data Availability

Reasonable requests for additional data will be considered by the authors. Raw data from the UK Transplant Registry are accessible upon application to NHS Blood and Transplant.

## Abbreviations

aROC: Area under the receiver operating characteristic curve
DIU: Donor insulin use
ICAM-1: Inter-cellular adhesion molecule-1
ICU: Intensive care unit
NHSBT: NHS Blood and Transplant
NRI: Net reclassification improvement
PTA/PAK: Pancreas transplant alone/pancreas after kidney transplant

## References

[1] Dean PG, Kukla A, Stegall MD, Kudva YC (2017). Pancreas transplantation. BMJ.

[2] Salvalaggio PR, Schnitzler MA, Abbott KC (2007). Patient and graft survival implications of simultaneous pancreas kidney transplantation from old donors. Am J Transplant.

[3] Humar A, Ramcharan T, Kandaswamy R, Gruessner RWG, Gruessner AG, Sutherland DER (2004). The impact of donor obesity on outcomes after cadaver pancreas transplants. Am J Transplant.

[4] Axelrod DA, Sung RS, Meyer KH, Wolfe RA, Kaufman DB (2010). Systematic evaluation of pancreas allograft quality, outcomes and geographic variation in utilization. Am J Transplant.

[5] Shapey IM, Summers A, Augustine T, Rutter MK, van Dellen D (2017). Pancreas transplantation: the donor’s side of the story. BMJ.

[6] Amaral PHF, Genzini T, Perosa M, Massarollo PCB (2015). Donor risk index does not predict graft survival after pancreas transplantation in Brazil. Transplant Proc.

[7] Finger EB, Radosevich DM, Dunn TB (2013). A composite risk model for predicting technical failure in pancreas transplantation. Am J Transplant.

[8] Rech TH, Crispim D, Rheinheimer J (2014). Brain death-induced inflammatory activity in human pancreatic tissue: a case-control study. Transplantation.

[9] Contreras JL, Eckstein C, Smyth CA (2003). Brain death significantly reduces isolated pancreatic islet yields and functionality in vitro and in vivo after transplantation in rats. Diabetes..

[10] Geer EB, Islam J, Buettner C (2014). Mechanisms of glucocorticoid-induced insulin resistance: focus on adipose tissue function and lipid metabolism. Endocrinol Metab Clin N Am.

[11] Aljada A, Ghanim H, Mohanty P, Kapur N, Dandona P (2002). Insulin inhibits the pro-inflammatory transcription factor early growth response gene-1 (Egr)-1 expression in mononuclear cells (MNC) and reduces plasma tissue factor (TF) and plasminogen activator inhibitor-1 (PAI-1) concentrations. J Clin Endocrinol Metab.

[12] Albacker T, Carvalho G, Schricker T, Lachapelle K (2008). High-dose insulin therapy attenuates systemic inflammatory response in coronary artery bypass grafting patients. Ann Thorac Surg.

[13] Ellger B, Langouche L, Richir M (2008). Modulation of regional nitric oxide metabolism: Blood glucose control or insulin?. Intensive Care Med.

[14] Novitzky D, Mi Z, Videla LA, Collins JF, Cooper DKC (2016). Hormone resuscitation therapy for brain-dead donors - is insulin beneficial or detrimental?. Clin Transpl.

[15] Shapey IM, Summers A, Yiannoullou P (2020). Donor insulin use predicts beta-cell function after islet transplantation. Diabetes Obes Metab.

[16] NHS Blood and Transplant (2012) Donation after brainstem death (DBD) donor optimisation extended care bundle. Available from: http://www.odt.nhs.uk/pdf/dbd_care_bundle.pdf. Accessed 13 May 2017

[17] Pencina MJ, D’Agostino RB, D’agostino RVR (2008). Evaluating the added predictive ability of a new marker: From area under the ROC curve to reclassification and beyond. Stat Med.

[18] Forbes S, Oram RA, Smith A (2016). Validation of the BETA-2 score: an improved tool to estimate beta cell function after clinical islet transplantation using a single fasting blood sample. Am J Transplant.

[19] Masson F, Thicoipe M, Gin H (1993). The endocrine pancreas in brain-dead donors. A prospective study in 25 patients. Transplantation..

[20] Mankad P, James A, Siriwardena AK, Elliott AC, Bruce JIE (2012). Insulin protects pancreatic acinar cells from cytosolic calcium overload and inhibition of plasma membrane calcium pump. J Biol Chem.

[21] Samad A, James A, Wong J (2014). Insulin protects pancreatic acinar cells from palmitoleic acid-induced cellular injury. J Biol Chem.

[22] Shapey IM, Summers A, Yiannoullou P (2019). Insulin therapy in organ donation and transplantation. Diabetes Obes Metab.

[23] Koh A, Senior P, Salam A (2010). Insulin-heparin infusions peritransplant substantially improve single-donor clinical islet transplant success. Transplantation..

[24] Maglione M, Ploeg RJ, Friend PJ (2013). Donor risk factors, retrieval technique, preservation and ischemia/reperfusion injury in pancreas transplantation. Curr Opin Organ Transplant.

[25] van Dellen D, Summers A, Trevelyan S, Tavakoli A, Augustine T, Pararajasingam R (2015). Incidence and histologic features of transplant graft pancreatitis: a single center experience. Exp Clin Transplant.

[26] Drognitz O, Obermaier R, Liu X (2004). Effects of organ preservation, ischemia time and caspase inhibition on apoptosis and microcirculation in rat pancreas transplantation. Am J Transplant.

[27] Keck T, Werner J, Schneider L, Gebhard M-M, Klar E (2003). Characterization of ischemia/reperfusion injury after pancreas transplantation and reduction by application of monoclonal antibodies against ICAM-1 in the rat. Surgery..

[28] Spetzler VN, Goldaracena N, Marquez MA (2015). Duodenal leaks after pancreas transplantation with enteric drainage - characteristics and risk factors. Transpl Int.

[29] Nath DS, Gruessner A, Kandaswamy R, Gruessner RW, Sutherland DE, Humar A (2005). Late anastomotic leaks in pancreas transplant recipients - clinical characteristics and predisposing factors. Clin Transpl.

[30] Knight RJ, Bodian C, Rodriguez-Laiz G, Guy SR, Fishbein TM (2000). Risk factors for intra-abdominal infection after pancreas transplantation. Am J Surg.

[31] Schaser K-D, Puhl G, Vollmar B (2005). In vivo imaging of human pancreatic microcirculation and pancreatic tissue injury in clinical pancreas transplantation. Am J Transplant.

[32] Maglione M, Oberhuber R, Cardini B (2010). Donor pretreatment with tetrahydrobiopterin saves pancreatic isografts from ischemia reperfusion injury in a mouse model. Am J Transplant.

[33] Preissler G, Eichhorn M, Waldner H, Winter H, Kleespies A, Massberg S (2012). Intercellular adhesion molecule-1 blockade attenuates inflammatory response and improves microvascular perfusion in rat pancreas grafts. Pancreas..

[34] Van den Berghe G, Wouters P, Weekers F (2001). Intensive insulin therapy in critically ill patients. N Engl J Med.

[35] Langouche L, Vanhorebeek I, Vlasselaers D (2005). Intensive insulin therapy protects the endothelium of critically ill patients. Blood..

[36] Vinkers MT, Rahmel AO, Slot MC, Smits JM, Schareck WD (2008). How to recognize a suitable pancreas donor: a Eurotransplant study of preprocurement factors. Transplant Proc.

[37] Blok JJ, Kopp WH, Verhagen MJ (2016). The value of PDRI and P-PASS as predictors of outcome after pancreas transplantation in a large European pancreas transplantation center. Pancreas..

[38] Mittal S, Lee FJ, Bradbury L (2015). Validation of the Pancreas Donor Risk Index for use in a UK population. Transpl Int.

[39] Ayami MS, Grzella S, Kykalos S, Viebahn R, Schenker P (2018). Pancreas donor risk index but not pre-procurement pancreas allocation suitability score predicts pancreas graft survival: a cohort study from a large German pancreas transplantation center. Ann Transplant.

